# Digital Image Disintegration Analysis: a Novel Quality Control Method for Fast Disintegrating Tablets

**DOI:** 10.1208/s12249-021-02080-0

**Published:** 2021-08-16

**Authors:** Osamah Malallah, Zara Rashid, Chee Lok Li, Abdulmalik Alqurshi, Mohamed A. Alhanan, Ben Forbes, Paul G. Royall

**Affiliations:** 1 Institute of Pharmaceutical Science, School of Cancer and Pharmaceutical Sciences, King’s College London, 150 Stamford Street, London, SE1 9NH UK; 2 Boots Pharmacy, The Brewery, 7 Romford, Essex, RM1 1AU UK; 3 The University of Hong Kong, Pok Fu Lam, Hong Kong; 4 Department of Pharmaceutics and Pharmaceutical Technology, College of Pharmacy, Taibah University, Al Madinah, Al Munawara Kingdom of Saudi Arabia

**Keywords:** Digital image disintegration analysis (DIDA), Fast disintegrating tablet, Quality control disintegration assay, 3D-printed disintegration vessel, Swelling, Freeze-dried pilocarpine HCl fast disintegrating tablets, Nurofen Meltlets, Imodium Instant

## Abstract

Measuring tablet disintegration is essential for quality control purposes; however, no established method adequately accounts for the timeframe or small volumes of the medium associated with the dissipation process for fast disintegrating tablets (FDTs) in the mouth. We hypothesised that digital imaging to measure disintegration in a low volume of the medium might discriminate between different types of FTD formulation. A digital image disintegration analysis (DIDA) was designed to measure tablet disintegration in 0.05–0.7 mL of medium. A temperature-controlled black vessel was 3D-printed to match the dimensions of each tablet under investigation. An overhead camera recorded the mean grey value of the tablet as a measure of the percentage of the formulation which remained intact as a function of time. Imodium Instants, Nurofen Meltlets and a developmental freeze-dried pilocarpine formulation were investigated. The imaging approach proved effective in discriminating the disintegration of different tablets (p < 0.05). For example, 10 s after 0.7 mL of a saliva simulant was applied, 2.0 ± 0.3% of the new pilocarpine tablet remained, whereas at the same time point, 22 ± 9% of the Imodium Instants had not undergone disintegration (temperature within the vessel was 37 ± 0.5°C). Nurofen Meltlets were observed to swell and showed a percentage recovery of 120.7 ± 2.4% and 135.0 ± 6.1% when 0.05 mL and 0.7 mL volumes were used, respectively. Thus, the new digital image disintegration analysis, DIDA, reported here effectively evaluated fast disintegrating tablets and has the potential as a quality control method for such formulations.

**Supplementary Information:**

The online version contains supplementary material available at 10.1208/s12249-021-02080-0.

## INTRODUCTION

Fast disintegrating tablets (FDTs) are dosage forms required to disintegrate or “melt” within a patient’s mouth without chewing or water intake (1). They have the advantages of simple administration, accurate dosing, rapid onset of action and avoidance of the first pass effect (2). The market for FDTs is large and is based on many established patented technologies and commercially available fast disintegrating tablet platforms, for example, Zydis®, Wowtab®, Ceaform®, Durasolv®, Flashtab®, Orasolv®, Sheaform® and Frosta® technologies (3,4). Pharmacopoeias provide imperfect guidance on the limits for the disintegration time of FDTs, and these times are typically measured using technology designed for normal release tablets. The disintegration time required for FDTs by the US Food and Drug Agency (FDA) is within 30 s compared to the European Pharmacopoeia time of 3 min (5,6), whereas both agree that normal release tablets must disintegrate between 15 and 30 min. For many categories of tablet, disintegration generally occurs as a prelude to dissolution, based on the definition of the disintegration process which can be explained as tablet breakdown into small granules, followed by disaggregation of the disintegrated granules (7). However, in specific cases, drug dissolution might happen quicker than drug disintegration (7). Consequently, there is a need to unify the pharmacopoeial disintegration time limits for FDTs and, more importantly, develop biorelevant techniques to measure this critical parameter accurately and routinely for quality control purposes.

The British and United States pharmacopoeia align their disintegration quality control tests for normal release tablets. They recommend apparatus with a basket-rack assembly containing 6 open-ended transparent tubes; each tube 75.0–80.0 mm long with an inside diameter of 20.7–26 mm. The base of each tube is formed from stainless-steel wire cloth, which has a plain square weave of 1.8–2.2 mm apertures. Each tablet is placed in the vessel, which is raised and lowered inside the basket at a frequency of 30 cycles per min. The recommended disintegration medium volume is 800 mL of water and a temperature of 37°C for the disintegration vessel. Although pharmacopoeias recommend using this technology for FDTs, the basket-rack assembly apparatus is not appropriate for measuring the disintegration times for such dosage forms. The endpoint or complete disintegration is achieved when all materials go through the mesh of the disintegration vessel. The basket-rack assembly is designed to simulate the disintegration of conventional tablets within the stomach, which is a very different environment from that found in the oral cavity of the mouth where FDTs disintegrate. In healthy individuals, the total volume of liquid available in the oral cavity is between 1 and 2 mL, typically replenished every minute, compared to the volume of the stomach of 300 to 500 mL during fasting and fed state, respectively (8,9). Also, fast disintegrating tablets are designed to help patients with swallowing difficulty, such as patients diagnosed with xerostomia, where a minimal volume is available for tablets to disintegrate ranging from 0.05 to 0.7 mL (9). The temperature within the mouth varies depending on location, for example, the sublingual region is typically 37°C, but the buccal area can range between 33 and 37°C, so the ability to control and operate at different temperatures needs to be considered in the design of new disintegration apparatus to represent the oral cavity (&,^10^–^12^). In addition, current methods are not optimal as the detection of the disintegration endpoint is a subjective measurement made by the operator.

Over the last 20 years, efforts have been made to design *in-vitro* procedures resembling the *in-vivo* disintegration environment of FDTs, such approaches have been based on texture analysis (TA) (13), CCD cameras (charged-coupled device) (14), Kyoto models (KYO) (15), charged-coupled devices (16), Tricorptesters (17) and the Aston test (18). There are limitations associated with all of the recent *in-vitro* disintegration assays. Firstly, the rate of measurement, typically data capture is too slow to gather enough measurements during the very rapid disintegration process. Secondly, the low discrimination sensitivity due to the large disintegration volumes used, which prevents testing formulations to narrow quality control specifications that are clinically relevant. The volumes utilised range from of 1 mL in the TA tests to the 400 mL used in the charged-coupled devices. Other disintegration assays such as Tricorptester and the Aston tests use a flow of disintegration medium of 6 mL/min and 10 mL/min, respectively (17,18). Such flows of disintegration medium overestimate the volume that a tablet will be exposed to in the timeframe in which it disintegrates. Finally, many of the new assays model the process of disintegration with the incorporation of a mechanical perturbation to replicate forces associated with mastication, such as the TA test (5 kg over 60 s) and the Aston test (50 g for a fixed time) (13,16,18). The disintegration measurement while applying a force on the formulation does not replicate the disintegration forces encountered during clinical use. The applied force will speed the disintegration process if the force is equal or more than that required force to break the interparticle forces within the formulation. For many FDTs, especially freeze-dried tablets, for example, Imodium Instants, the advice to patients is not to chew. The typical disintegration rates upon placing the solid monolith in the mouth are so quick, within seconds, that modelling mastication as part of any disintegration assay is redundant.

The aim of this study was to develop a new disintegration method to overcome current shortcomings and allow better quality control of FDTs. Any new *in-vitro* analysis procedure must have the ability to discriminate between the disintegration of a broad range of the FDT’s while simulating the conditions in which they are used clinically, i.e., conditions such as xerostomia where a limited volume of liquid is available, and temperature should be maintain within the range found in the oral cavity. In this study, we hypothesised that digital imaging provides an opportunity to make accurate and frequent measures of disintegration in a bespoke low volume temperature-controlled vessel to provide an assay that will discriminate between different types of FDT formulation. A set of training formulations was chosen to represent a variety of fast disintegration technologies; it included a highly porous dosage form for buccal administration; a compact freeze-dried tablet designed to be placed on the buccal area and a directly compressed tablet incorporating super disintegrating excipients.

## MATERIALS AND METHODS

### Tested Formulations

Loperamide HCl licensed as Imodium Instant (McNeil Products Limited) and ibuprofen licensed as Nurofen Meltlets (Reckitt Benckiser Healthcare UK Ltd) were used as model products. A novel developmental freeze-dried fast disintegrating pilocarpine HCl tablet (19) made up the training set of three.

### Development of Digital Image Disintegration Analysis

#### Construction of Disintegration Vessel

The disintegration vessel was 3D-printed, designed using Autodesk® 3ds Max® Design version 2018 (Autodesk Inc., NY, CA, USA). The design was then exported to the 3D printer Makerbot 5th Gen Mini+ (MakerBot Industries, Brooklyn, NY, USA). The disintegration vessel for each formulation was printed using graphite black PLA (polylactic acid) filament (Monster Fil, Batch NO 1800889). The printing settings were extrusion at 230°C and 20°C platform temperature with raft option activated. Bespoke 3D-printed disintegration vessels were produced for Nurofen Meltlets, Imodium Instants and freeze-dried pilocarpine HCl tablets.

#### Temperature Control

The temperature of both the 3D-printed disintegration vessel and the disintegration medium, 0.05 and 0.7 mL phosphate buffer saline (PBS, pH 7), under assay conditions, was measured at 33°C and 37°C to validate deviation < 0.5°C from the target temperature. In real life, tablet administration might affect the temperature of oral cavity. However, subsequent investigations are required to study the effect of such change on tablet disintegration. The 3D-printed disintegration vessel was fitted at the neck of a Duran bottle filled with water. The 3D-printed disintegration vessel containing the PBS was then placed in a water bath composed of two compartments (Grant Instruments Ltd, Ser No: UJ1929001), to control the temperature of both the disintegration vessel and PBS separately. To further maintain the target temperature of the system, a layer of high-performance insulation sheeting encased in aluminium (YBS insulation) was used to cover the open surface of the water bath.

The temperature of the disintegration vessel and disintegration medium was measured using a digital thermometer (a micro-thermocouple (TC Ltd Uxbridge UK, CC: 401-324 specification: Welded Tip PFA with Plug)) and a temperature logger (TC Ltd Uxbridge UK, CC: 753-671, model: YC-747U, Multi Thermocouple Type Indicator). The temperature of the digital thermocouple was validated following two methodologies. The first method was by filling two containers, once with pre-heated hot water and the other with cold water. The temperature of the water in the two containers was measured by a digital thermometer and also by a conventional thermometer. The second method was measuring the temperature of ice water, an accepted approach in thermal analysis where water and ice in equilibrium temperature validation of an empty 3D-printed disintegration vessel.

The ability to maintain the target temperature, in these experiments 33°C or 37°C, of both the empty 3D-printed disintegration vessel and the PBS for use as disintegration medium over 250 s was important for analysis validation. The temperature was recorded by placing a micro-thermocouple at the centre of the disintegration vessel or in the disintegration medium, n = 3.

##### Temperature Validation of 3D-Printed Disintegration Vessel Loaded with 0.05 or 0.7 mL PBS

Small volumes medium transferred between vessels are vulnerable to heat dissipation and thus a reduction in temperature (20). The ability to maintain the temperature of the disintegration medium during transfer of the required volume of the disintegration medium to the 3D-printed disintegration vessel was investigated. The temperature of the PBS solution and the 3D-printed kit containing 0.05 or 0.7 mL of PBS at 33°C and 37°C was measured over 250 s for both Imodium Instant and freeze-dried pilocarpine HCl and 200 s for Nurofen Meltlets. The temperature of the specified volumes was measured in three different occasions, n = 3.

##### Temperature Validation of 3D-Printed Disintegration Vessel During the Disintegration Process

The ability to maintain the temperature of the disintegration vessel during the disintegration process of the tested formulations was investigated. This validation was conducted by measuring the temperature using a thermocouple placed at the centre of the 3D-printed disintegration vessel. The tested tablet was then placed in the 3D-printed disintegration vessel, and the required volume of PBS then dispensed over the tablet using Gilson pipette, n = 3.

#### Measurement of Camera Noise

The 3D-printed disintegration vessel was positioned under a digital USB microscopic camera purchased from MixMart company. The camera specifications were 2MP video camera 500× zoom and imaging (1600 × 1200) pixel. The distance between the camera and the 3D-printed disintegration vessel was adjusted to observe the tested formulations without zooming. A video for (1) disintegration media only (background); (2) tested tablet only and (3) tablet disintegration was recorded over 250 s. The video was converted to images, which was then analysed using a Java-based image processing program to measure the mean grey value (MGV) called ImageJ software.

#### Disintegration Profile

**Fig. 1:**
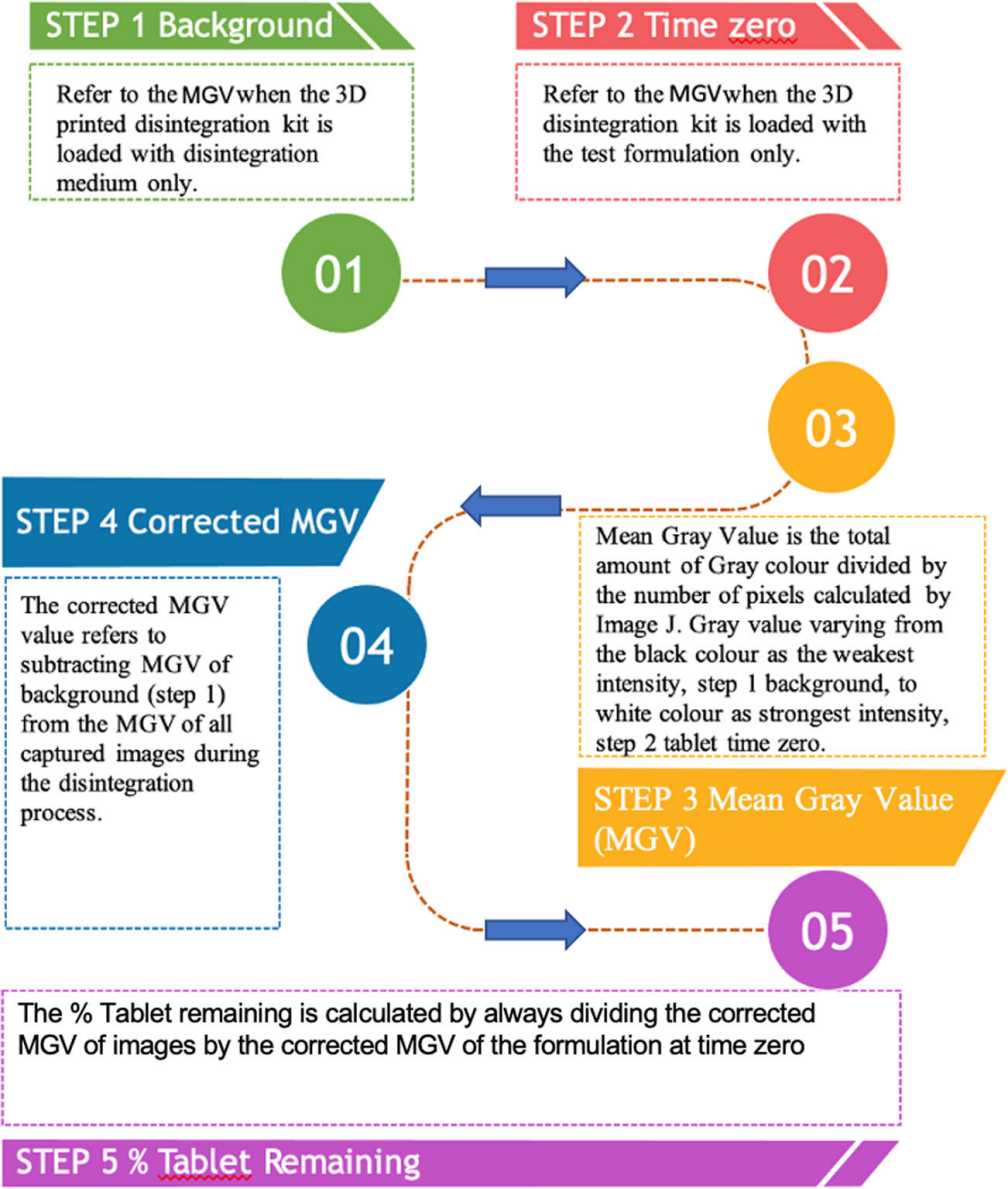
A flowchart describing the process of data collection and analysis of tablet disintegration measurements through digital imaging. Measurements of mean grey values (MGVs) were determined and calculated using ImageJ. High-resolution images were collected at a rate of 10 images per second. MGV was determined based on a fixed area covering only the test formulation (n = 3).

The MGV gave a measure of the amount of white material in the disintegration vessel. The background MGV, determined for the 3D-printed disintegration vessel containing only the disintegration medium, was subtracted from each MGV measured to adjust for background noise. Calculated MGV of all images for a disintegration test were plotted against time to generate a disintegration profile of the test tablet. MGV were converted to a percentage of the dosage remaining, using the MGV of the full tablet as 100% (Fig. 1).

## RESULTS

### Digital Image Disintegration Vessel and 3D-Printed Disintegration Vessel

**Fig. 2:**
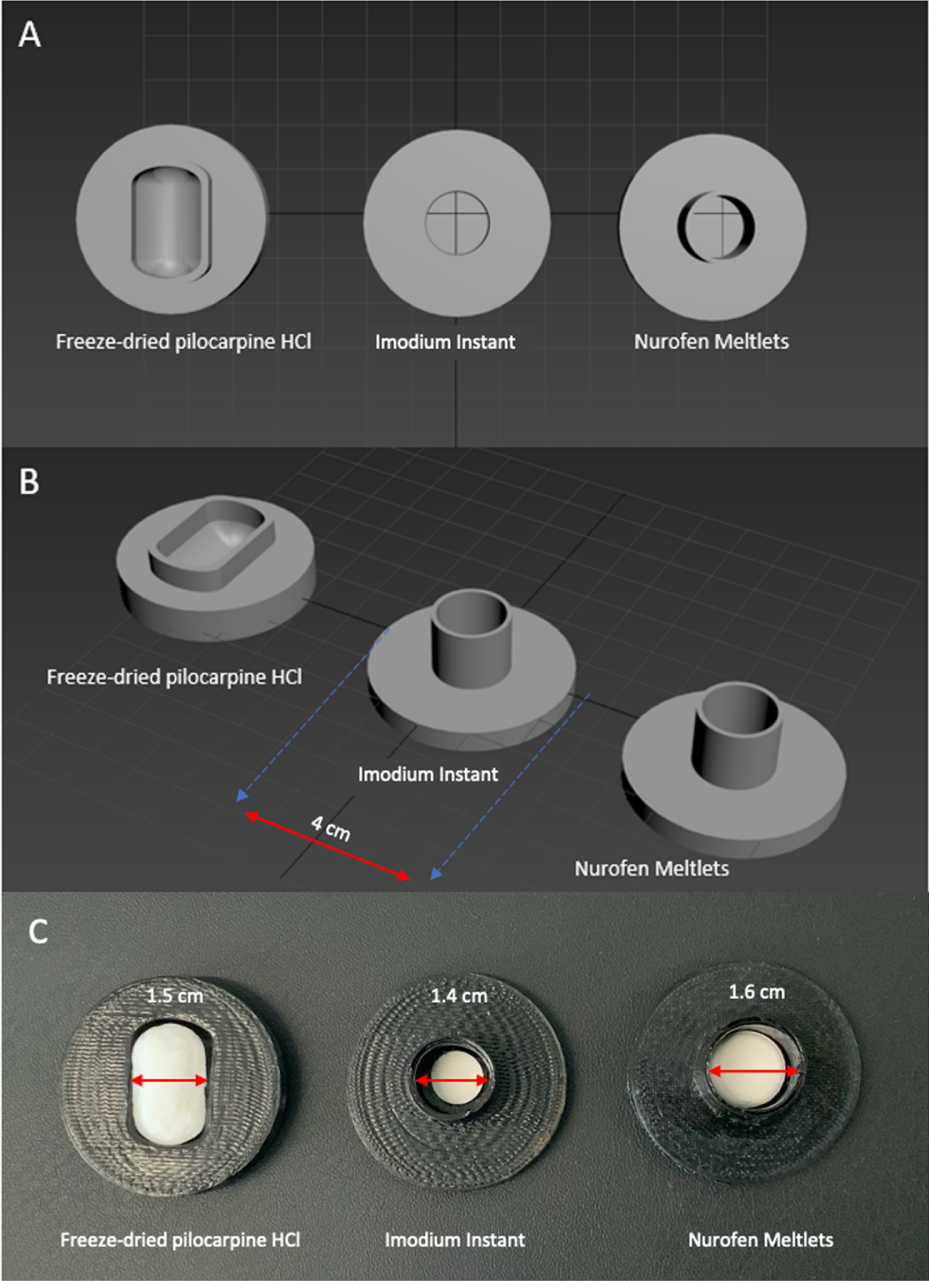
3D-printed disintegration vessels for Imodium Instants, freeze-dried pilocarpine HCl and Nurofen Meltlets rendered images of **A** top view, **B** side view and **C** photograph of tested formulation loaded in designed 3D-printed disintegration vessel.

The 3D-printed disintegration vessels were manufactured in dimensions that provided a close fit, allowing enough space to load the tablet into the vessel without requiring excessive force thus avoiding disruptions to the tablet’s structure and also avoiding loading damage, Fig. Fig. 2. The choice of the colour used for the 3D printing gave a good contrast between the black colour of the kit and the white colour of the tested formulations, Fig. Fig. 2C.

**Fig. 3:**
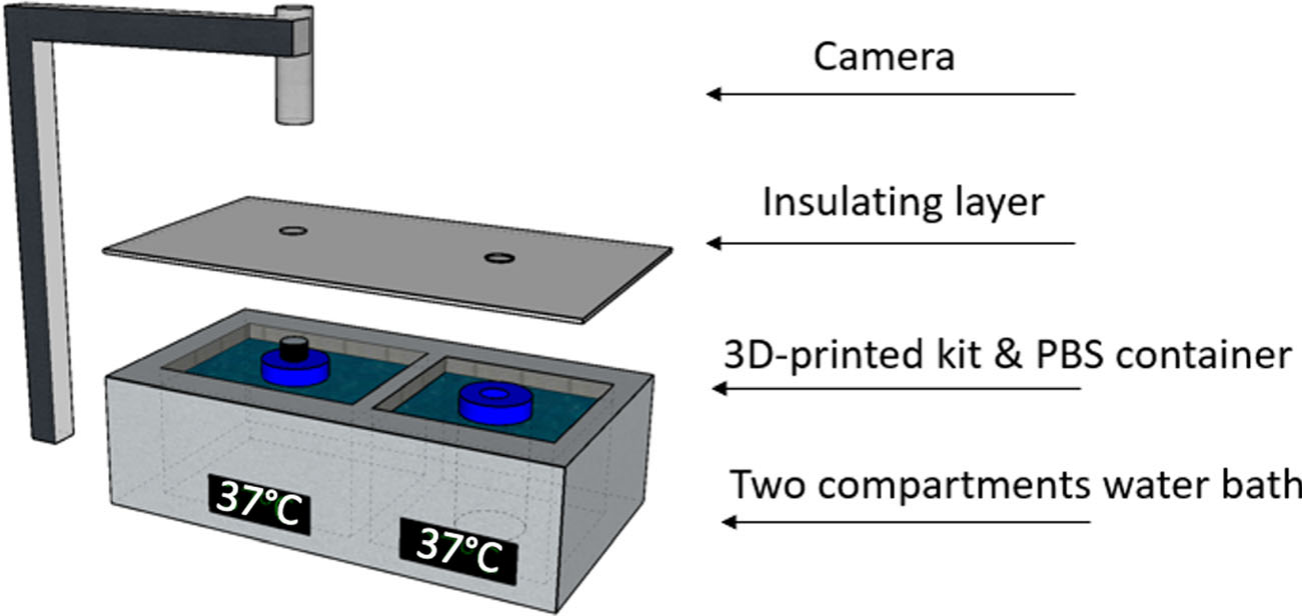
Schematic diagram for the digital image disintegration analysis. The disintegration vessel was 3D-printed graphite black PLA filament to produce contrast for the tablet. The camera was attached to a stand to be positioned on the top of the 3D-printed kit that was fitted on the neck of Duran bottle. An insulating layer was used to cover the top surface of the water bath for further temperature control. The disintegration of the tablet was monitored as the mean grey value using an image analyser.

A simple DIDA system was constructed for ease of operation while maintaining the accuracy required for temperature control, image capture and disintegration medium volume. The camera was attached to a stand with a fixed distance between the camera and the disintegration vessel to minimise variation between assays. Placing the disintegration vessel and disintegration medium in the two-compartment water bath along with the application of the insulating layer created a highly temperature-controlled environment for disintegration studies, Fig. 3 and Table I.

**Table I:** Temperature Validation of 33 and 37°C of the 3D-Printed Disintegration Vessel When It Is Empty, Loaded with 0.05 or 0.7 mL of PBS, and During the Disintegration Process of Imodium Instant Tablet and Pilocarpine HCl Freeze-Dried Tablet. The Temperature Was Measured Each Time over 250 s, with a Temperature Reading Frequency of 1 Reading/s. The Data Illustrated Shows the Mean Temperature at the Start and at the End of the Disintegration Process, ± Symbol Represents Standard Deviation Between Readings, n = 3.

Temperature validation		Mean temperature at the start (°C)	Mean temperature at the end (°C)
Imodium Instant	3D-printed kit at 33°C	33.13 ± 0.12	33.22 ± 0.21
	3D-printed kit with 0.05 mL PBS at 33°C	32.74 ± 0.25	32.93 ± 0.04
	3D-printed kit with tablet and 0.05 mL PBS at 33°C	32.85 ± 0.14	32.98 ± 0.02
	3D-printed kit with 0.7 mL PBS at 33°C	33.16 ± 0.23	32.80 ± 0.31
	3D-printed kit with tablet and 0.7 mL PBS at 33°C	33.27 ± 0.34	33.05 ± 0.47
	3D-printed kit at 37°C	37.29 ± 0.45	37.03 ± 0.06
	3D-printed kit with 0.05 mL PBS at 37°C	36.76 ± 0.25	37.31 ± 0.24
	3D-printed kit with tablet and 0.05 mL PBS at 37°C	37.05 ± 0.21	37.16 ± 0.31
	3D-printed kit with 0.7 mL PBS at 37°C	36.89 ± 0.18	36.82 ± 0.30
	3D-printed kit with tablet and 0.7 mL PBS at 37°C	36.93 ± 0.13	36.68 ± 0.43
Freeze-dried pilocarpine HCl	3D-printed kit at 33°C	32.97 ± 0.02	33.16 ± 0.29
	3D-printed kit with 0.05 mL PBS at 33°C	33.23 ± 0.35	32.96 ± 0.39
	3D-printed kit with tablet and 0.05 mL PBS at 33°C	32.62 ± 0.06	32.58 ± 0.15
	3D-printed kit with 0.7 mL PBS at 33°C	32.79 ± 0.13	32.43 ± 0.09
	3D-printed kit with tablet and 0.7 mL PBS at 33°C	32.86 ± 0.13	32.64 ± 0.21
	3D-printed kit at 37°C	37.08 ± 0.23	36.72 ± 0.15
	3D-printed kit with 0.05 mL PBS at 37°C	36.86 ± 0.17	36.58 ± 0.31
	3D-printed kit with tablet and 0.05 mL PBS at 37°C	36.98 ± 0.07	36.78 ± 0.37
	3D-printed kit with 0.7 mL PBS at 37°C	37.04 ± 0.20	36.90 ± 0.11
	3D-printed kit with tablet and 0.7 mL PBS at 37°C	36.81 ± 0.13	37.13 ± 0.45

*PBS*, phosphate buffer saline

### Validation of Temperature Control and Camera Noise

The temperature was validated in different configurations to ensure the target temperature was achieved and maintained during the disintegration of the tested formulations. Target temperatures of 33°C and 37°C of the 3D-printed disintegration vessel were measured when the disintegration vessel was empty, loaded with volumes of 0.05 or 0.7 mL of PBS and loaded with tested formulations, Table I. The MGV values were validated for different 3D-printed disintegration vessel, different formulations and different disintegration media volume to ensure that the reduction of the percentage tablet remaining is due to an actual disintegration process of the tablets rather than background noise generated by poor rate of picture acquisition of the camera, Appendix A.

The temperature validation results for Nurofen Meltlet showed that the target temperature of 37°C was well controlled within the range of ± 0.55°C, as illustrated in Table II.

**Table II:** Multiple Stages Temperature Validation 37°C of the 3D-Printed Disintegration Vessel When Loaded with 0.05 or 0.7 mL of PBS and During the Disintegration Process of Nurofen Meltlet. ± Symbol Represents Standard Deviation Between Measurements, over 250 s with Temperature Reading Frequency of 1 Reading/s. Each Temperature Was Validated Three Times.

Temperature validation	The mean temperature at the start (°C)	Mean temperature at the end (°C) ± Standard deviation (°C)
3D-printed kit with 0.05 mL PBS at 37°C	37.52 ± 0.00	37.51 ± 0.00
3D-printed kit with tablet and 0.05 mL PBS at 37°C	36.56 ± 0.00	37.47 ± 0.05
3D-printed kit with 0.7 mL PBS at 37°C	36.94 ± 0.00	36.93 ± 0.05
3D-printed kit with tablet and 0.7 mL PBS at 37°C	37.13 ± 0.42	37.44 ± 0.09

*PBS*, phosphate buffer saline

### Formulation Screening

**Fig. 4:**
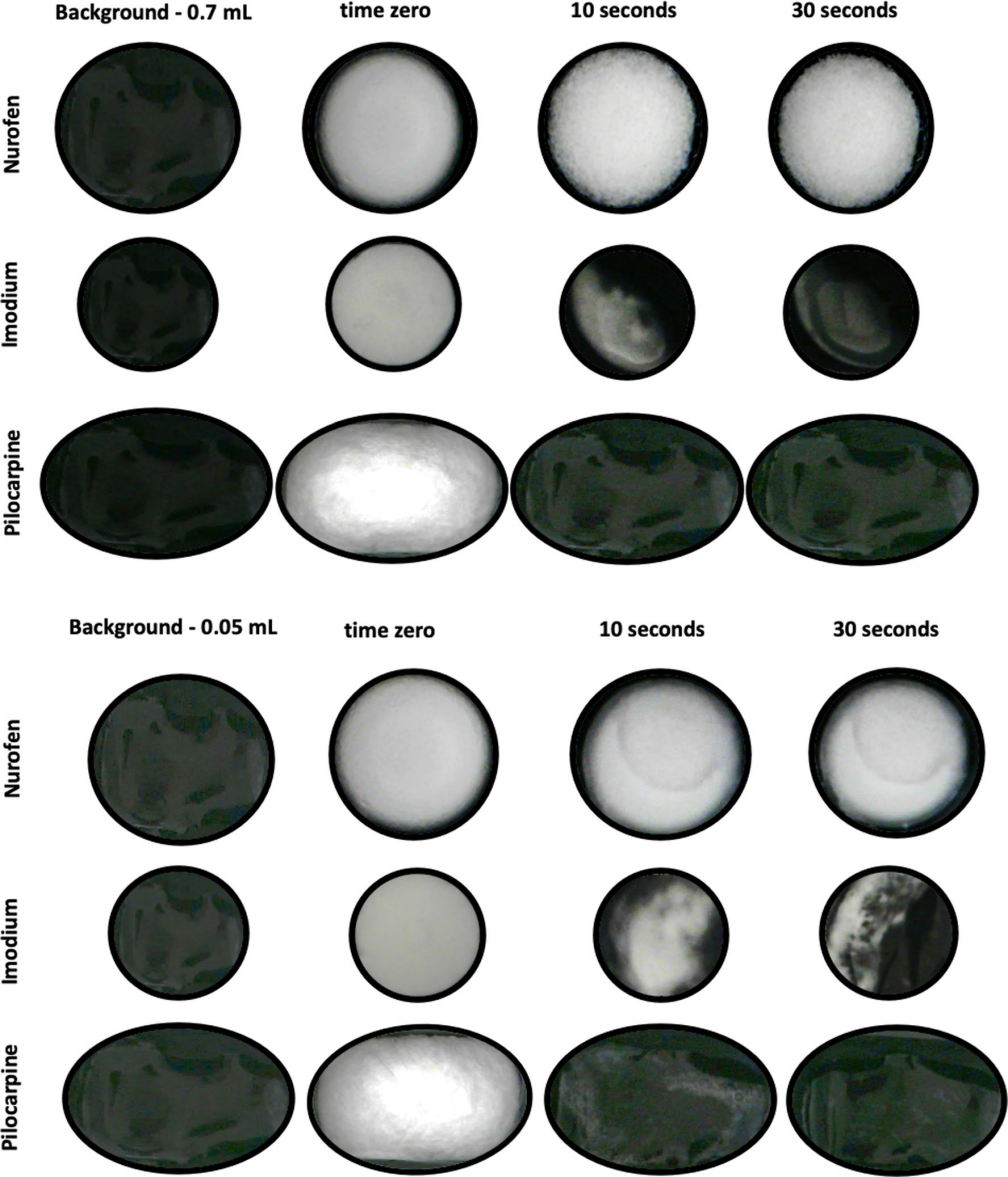
Photos captured by DIDA during the disintegration process of freeze-dried pilocarpine HCl, marketed Imodium Instants and Nurofen Meltlets, which was labelled as pilocarpine, Imodium and Nurofen, respectively. The captured images were for the background (empty disintegration vessel), formulations at time zero (formulation loaded into the disintegration vessel without disintegration media) and time points of 10 s and 30 s at 37°C using different sets of volumes, 0.7 mL and 0.05 mL, of phosphate buffer.

**Fig. 5:**
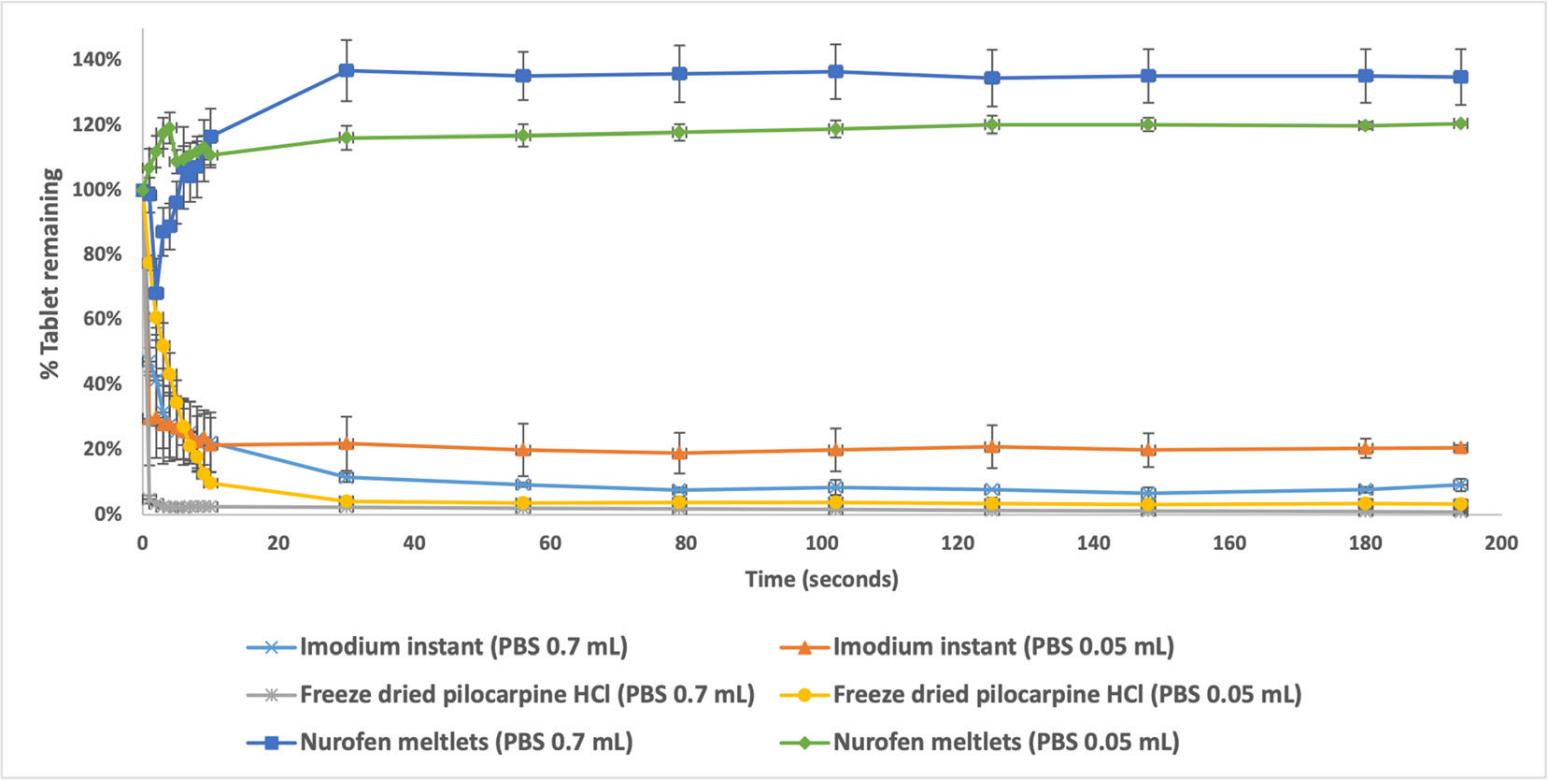
The disintegration curves of freeze-dried pilocarpine HCl, marketed Imodium Instants and Nurofen Meltlets 37°C using 0.7 mL and 0.05 mL of phosphate buffer as disintegration medium. *Values above 100% represent swelling.* The error bars represent the standard deviation between samples.

The disintegration of freeze-dried pilocarpine HCl, Imodium Instants and Nurofen Meltlets was tested first using 0.05 mL and 0.7 mL at a temperature of 37°C over 200 s, Figs. 4 and 5. An example of data analysis to generate percentage tablet disintegration was added to supplementary data, Appendix B. Overall, the disintegration profiles of Nurofen Meltlets showed an increase in the percentage of tablet remaining over time. The disintegration results of Nurofen Meltlets tablets showed a percentage recovery of 120.7 ± 2.4% and 135.0 ± 6.1% when 0.05 mL and 0.7 mL volumes were used, respectively, *p*-value 0.1305, Fig. 5. These results were supported by the digital images taken during the disintegration process where an increase in the surface area of the tablets was recorded with 0.7 mL PBS and with 0.05 mL PBS, Fig. 4. Both disintegration profiles and the captured images indicated that the Nurofen Meltlets tablets swelled over time upon the absorption of the disintegration media. Formulation swelling was assumed to be related to the hypromellose excipient present. Thus, tablets composed of 100% (w/w) hypromellose were formulated and tested with 0.7 mL to monitor the swelling of the tablet over time. Adding 0.7 mL PBS to hypromellose tablets showed an increase in the height of the tablet from 0.50 ± 0.00 to 0.7 ± 0.05 cm over 30 min, *p*-value 0.0377, Appendix C.

The digital image disintegration analysis was successful in discriminating between the disintegration of the three different formulations. Having such a system is important where the disintegration behaviour of orally disintegrating tablets can be categorised into instant (freeze-dried pilocarpine HCl), fast (Imodium Instants) and prolonged disintegration (Nurofen Meltlets) orally disintegrating tablets. Understanding the factors contributing to the disintegration process of orally disintegrating tablets is essential when investigating performance aspects of the formulation during the early stages of development. For example, data recorded by the new DIDA approach showed that in limited disintegration volumes, the swelling properties of hypromellose dominate to the detriment of tablet disintegration. Such an observation has not been previously reported as currently available methods incorporate much larger volumes of disintegration media. Further investigation of the role of volume and temperature on the instant (freeze-dried pilocarpine) and the fast (Imodium Instants) disintegrating tablets was possible from the data generated by the DIDA approach.

### Impact of Different Temperatures and Disintegration Medium Volume on the Disintegration of Imodium Instant Tablets and Freeze-Dried Pilocarpine HCl

#### Imodium Instant

**Fig. 6:**
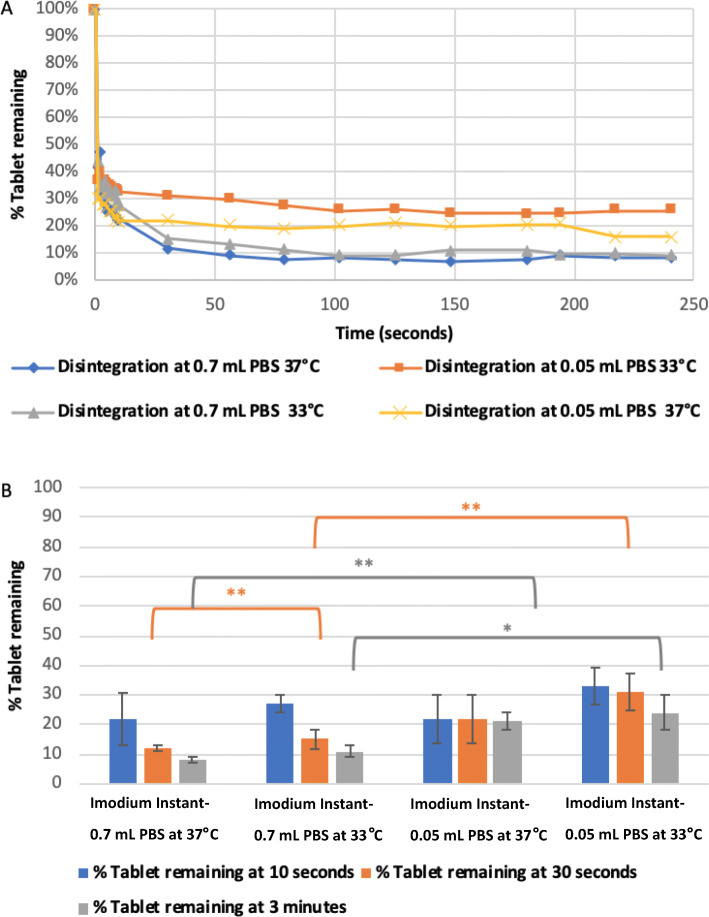
The disintegration curve of marketed Imodium Instant at 33°C and 37°C using 0.7 mL and 0.05 mL of phosphate buffer as disintegration medium. **A** % tablet remaining over 250 s. **B** % tablet remaining at time points of 10 s, 30 s and 3 min. The disintegration vessel was 3D-printed kit was specifically made based on the dimensions of licensed Imodium Instant® tablets. The error bars represent the standard deviation between samples and the symbol. *, **, *** represent P ≤ 0.05, P ≤ 0.01 and P ≤ 0.001, respectively.

Alignment of the generated disintegration profiles of Imodium Instant tablets at different temperatures and volumes of disintegration media showed that the percentage recovery value (% tablet not disintegrated) is lower, i.e., faster disintegration of the formulation achieved, at disintegration volume of 0.7 mL PBS, Fig. 6A. For example, evaluating the disintegration process at the end of 3 min at a temperature of 37°C using 0.05 and 0.7 mL PBS achieved a significant difference in the percentage tablet remaining of 21 ± 3% and 8 ± 1%, respectively, (*p-*value of 0.0062), Fig. 6B. In general, measuring tablet disintegration at a different temperature of 33°C or 37°C did not have a significant effect on the percentage of tablet remaining except for one combination (30 s with 0.7 mL PBS) which achieved a value of 12 ± 1% and 17 ± 3% at 37°C and 33°C, respectively, (*p-*value of 0.0026), Fig. 6B. In all cases, Imodium Instant tablets showed incomplete disintegration where a small amount of the material remained (not disintegrating), the lower the temperature and the disintegration volume, the higher amount of material left.

#### Freeze-Dried Pilocarpine HCl

**Fig. 7:**
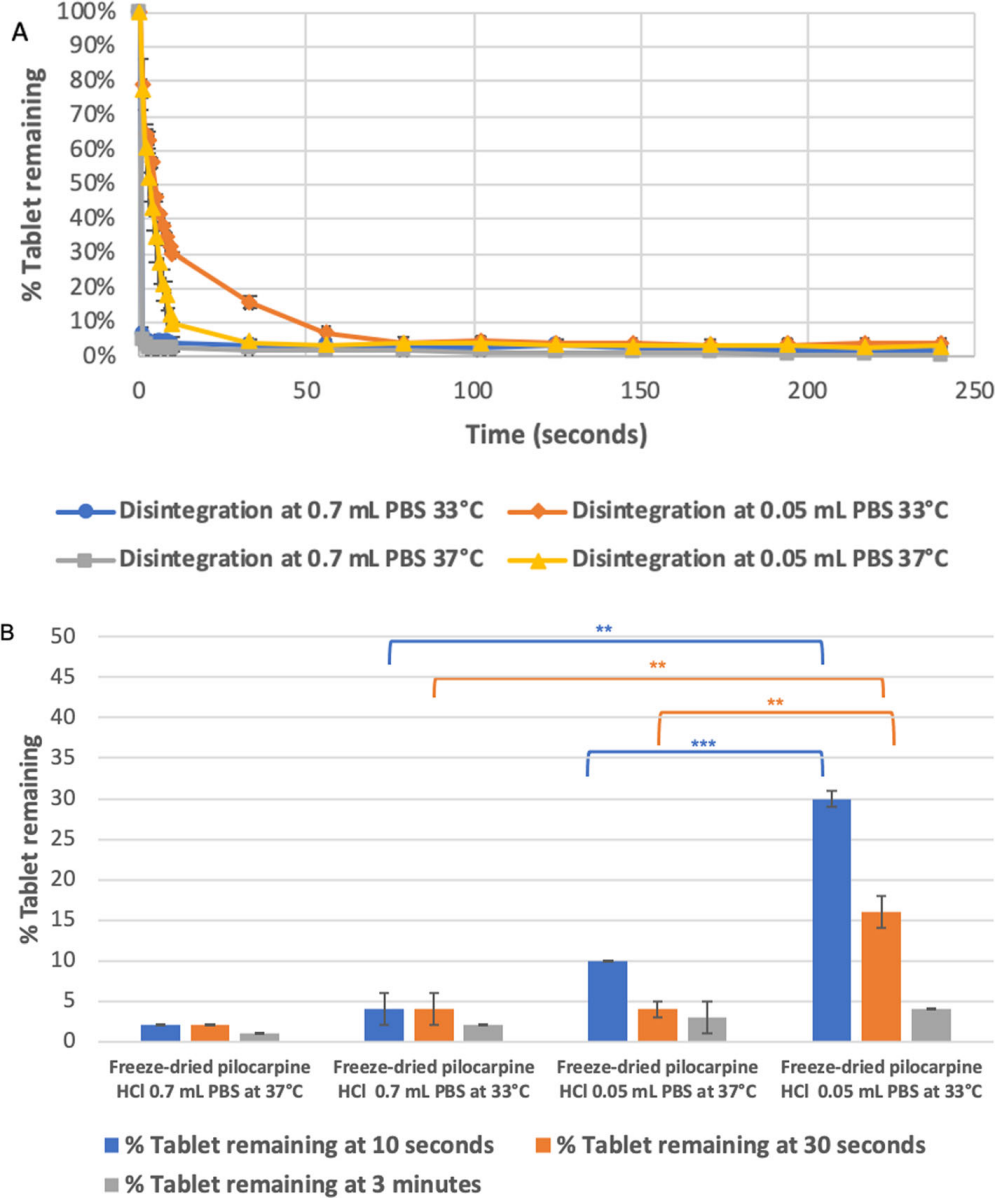
The disintegration curve of pilocarpine HCl FDT at 33°C and 37°C using 0.7 mL and 0.05 mL of phosphate buffer as disintegration medium. **A** % tablet remaining over 250 s. **B** % tablet remaining at time points of 10 s, 30 s and 3 min. The disintegration vessel was 3D-printed kit was explicitly made based on the dimensions of pilocarpine HCl FDT. The error bars represent the standard deviation between samples and the symbol. *, **, *** represent P ≤ 0.05, P ≤ 0.01 and P ≤ 0.001, respectively.

**Fig. 8:**
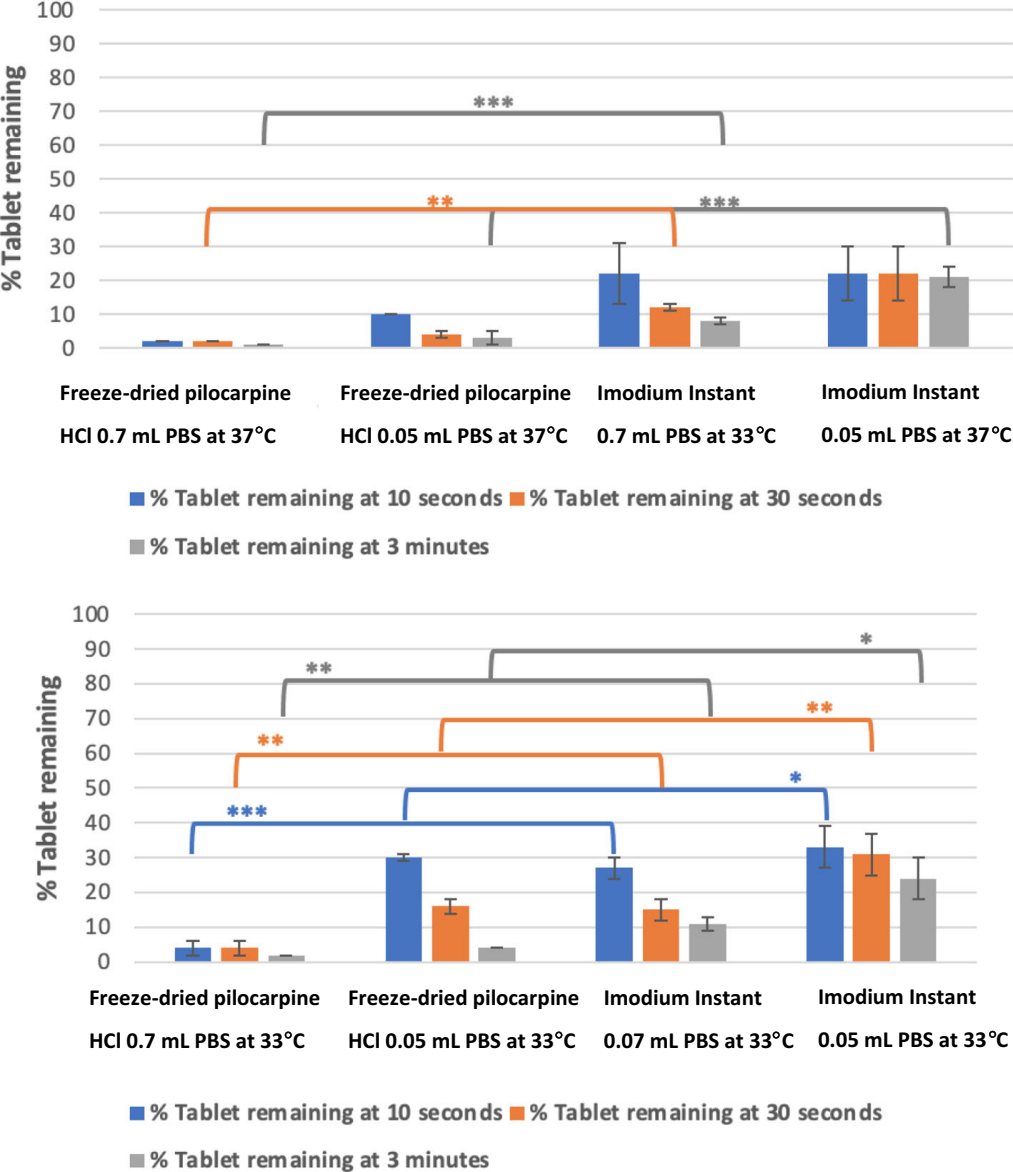
Comparison of % tablet remaining of pilocarpine HCl OFD and marketed Imodium Instant® at time points of 10 s, 30 s and 3 min using 0.7 mL and 0.05 mL of phosphate buffer as disintegration medium. **A** % tablet remaining at 37°C. **B** % tablet remaining at 33°C. The error bars represent the standard deviation between samples and the symbol. *, **, *** represent P ≤ 0.05, P ≤ 0.01 and P ≤ 0.001, respectively.

The effect of different temperatures of 33°C and 37°C and disintegration volumes of 0.05 mL and 0.7 mL PBS on the disintegration of freeze-dried pilocarpine HCl at time points of 10 s, 30 s and 3 min was evaluated using DIDA, Fig. 7. The disintegration profile of the formulation under different tested conditions showed a reduction in the percentage tablet recovery over time, Fig. 7A. DIDA was sensitive to changes in the system temperature and changes in the volume of the disintegration medium during the disintegration process of the freeze-dried pilocarpine HCl tablet. Comparing the percentage tablet remaining at the end of 10 s when the system temperature at 33°C was fixed while changing the disintegration volume from 0.05 to 0.7 mL of PBS recorded a value of 30.1 ± 1.5% and 4.0 ± 2.0%, respectively, (*p*-value of 0.0032), Fig. 7B. Similarly, the percentage tablet remaining of 30.1 ± 1.5% and 10.0 ± 1.2% was measured when the system temperature was changed from 33 to 37°C using a fixed volume of 0.05 mL PBS, respectively, (*p*-value of 0.0003), Fig. 7B. Comparing the generated disintegration results of Imodium Instant tablets and pilocarpine HCl freeze-dried tablets clearly showed that the analysis was sensitive to the change of disintegration time due to the effect of temperature, disintegration media volume and tablet composition, see Fig. 8 for further values and statistical evidence.

## DISCUSSION

The novel imaging approach reported in this study was able to quantitatively record the disintegration of a set of representative FDTs. Such measurements are not possible with the current tests recommended by the BP, EMA and USP. The DIDA approach used volumes of media which are closer to the *in-vivo* situation. The choice of buffer and pH used here in the development of DIDA was supported by recent studies where water or phosphate-buffered saline was used as a disintegration media to mimic the pH range observed in the oral cavity of healthy patients, i.e., between 6.8 and 7.2 (21–24). The DIDA approach is inherently flexible, so the type of disintegration medium may be changed easily, allowing the exploration of the impact of different simulants of human saliva. This study used PBS as the medium and investigated the effect of volumes, 0.05 mL and 0.7 mL, representing the disintegration environment in the mouth of patients with xerostomia at two temperatures, selected to represent temperature variation during day and night (9,12).

The FDT formulations investigated in this study were Imodium Instants, Nurofen Meltlets and a formulation of freeze-dried pilocarpine HCl tablets under development for the treatment of patients with xerostomia. Drug containing films are a different class of dosage form and were not considered in this study. Although films are not designed to disintegrate as quickly as FDT’s, the current DIDA approach could be adapted for such systems, whereby the colour of the 3D-printed vessel could be optimised to increase contrast with the opaque film and the design for the vessel could be easily modified. However, film dosage forms are unsuitable in patients experiencing mucosal sensitivity, for example, those diagnosed with xerostomia (9). The FDTs selected for this study included two of the formulations that are commercially available but showed extremely different responses that were only detected by using the small volume of disintegration medium applied in DIDA. The tested formulations displayed three distinct outcomes. Imodium Instants only partially disintegrated, pilocarpine tablets disintegrated quickly and completely and Nurofen Meltlets swelled rather than disintegrated (Figs. 4 and 5). The colour selected for the 3D-printed disintegration vessel allowed qualitative differential measurements between the disintegration vessel’s black background and the tested formulation’s white. The 3D printing approach for the disintegration vessel provided a flexible platform, as by simply amending the printing software, vessels cantering for different tablet size and shape were manufactured. Besides, the use of 3D-printed disintegration vessel enhances the accuracy of the measured % of tablet remaining over the disintegration process. It has the required area and depth to contain the disintegrated formulation to allow the camera to capture the whole of tablet’s disintegration process. Factors such as light intensity during the measurement were kept constant as varying the light intensity might affect the measured MGV of captured images during the disintegration process. Similarly, the distance between the camera and the tested formulation was kept constant during all experiments.

When DIDA was used to study Nurofen Meltlets, the MGV increased upon interaction with the small volumes of aqueous media applied. This was attributed to the used of hypromellose as an excipient in the formulation matrix. Hypromellose is hydroxypropyl methylcellulose polymer (HPMC) with different molecular weights based on the attached hydrocarbon chains (25). Hypromellose swells rather than disintegrates and develops a gel layer on the surface of a tablet that can limit drug diffusion out of the matrix (26,27). Water penetrates into the pores within the solid excipient present in the meltlets and has a high affinity for the hypromellose (28). The hydration of the hypromellose polymer within the pores causes swelling and the formation of a turbid gelled system (29), which increases contrast and thus the MGV of the image taken of the Meltlet. When the amount of liquid water is limited, the dispersion of this viscous gel is not observed as would have been the case if the aqueous media had been in excess (30).

The gelling mechanism resulted in an apparent increase in the observed % of the tablet remaining over time instead of the expected decrease, in conjunction with an increase in the height of the formulation (Appendix B). Thus, the calculated percentage recovery of Nurofen Meltlets tablets, at the end of the experiment, was 120.6 ± 3.4% and 135.0 ± 8.6% when 0.05 mL and 0.7mL volumes were used, respectively (Fig. 5). To further confirm that the observations of swelling behaviour of Nurofen Meltlets were related to the presence of hypromellose, tablets composed of 100% (w/w) of hypromellose were manufactured and exposed to small amounts of aqueous medium. The results confirmed that the hypromellose tablets swelled upon the addition of 0.7 mL PBS, producing an increase of tablet height, as illustrated in Appendix C. The water present causes the polymer chains of hypromellose to uncoil and extend, causing the disruption of inter and intra polymer hydrogen bonding and thus allowing an increase in available sites for further hydrogen bonds with water molecules resulting in material swelling (31). The conventional disintegration methods did not pick up the swelling mechanism during tablet disintegration in large disintegration volume of 800 mL, Appendix D. In the DIDA, when the amount of disintegration medium was reduced to mimic the *in-vivo* situation, Nurofen Meltlets swelled but did not disintegrate. Such formulations pass the current disintegration tests because of the large amount of medium employed. And this situation is compounded by the non-specific directions of British pharmacopoeia and other pharmacopoeias in terms of disintegration. For example, complete disintegration is defined by the British pharmacopoeia as “Any residue of the unit, except fragments of an insoluble coating or capsule shell, remaining on the screen of the test apparatus or adhering to the lower surface of the discs, if used, is a soft mass having no palpably firm core” (32). Thus, the endpoints of current pharmacopoeia approaches are ambiguous.

The patient leaflet of Nurofen Meltlets instructs patients to place the tablet on the tongue, allow it to dissolve and then swallow it, without the need of water or chewing. If a patient with xerostomia follows these instructions, they might suffer complications such as choking as the findings from the DIDA approach show that Nurofen Meltlets may not disintegrate in the limited amount of saliva, 0.05 mL, typically available to such patients (9).

Both the freeze-dried pilocarpine and Imodium Instant FDT are embedded in freeze-dried matrices of D-mannitol, gelatin and sodium bicarbonate (33). Therefore, it was expected that the disintegration time of these formulations would be similar. However, the DIDA found that Imodium Instants had longer disintegration time in comparison to pilocarpine FDT (Figs. 6 and 7). The disparity between the disintegration profiles arises from differences in the ingress of disintegration medium, which is the first step in the disintegration process for both formulations. Factors that affect the ingress of disintegration medium are the following: (1) polymer-drug interactions (34); (2) pore closure (35) and (3) the distribution of the formulation components within the tablet, for example, burst release happens if the drug or the disintegrating agent is close to the surface and thus accessible to disintegration media (36).

Higher gelatin to D-mannitol ratio in tablets has been observed to result in a longer disintegration time (37,38), although the basis of this effect was not investigated. The disintegration process starts by wetting (39) and FDT will be wetted by saliva and moisture absorbed from air, which has a humidity of 90 to 95% in the oral cavity (18). Moisture uptake is enhanced by higher gelatin concentrations in edible films due to the amorphous nature of this polymer (40). Similar behaviour can be assumed for Imodium Instant formulations which have a high ratio of gelatin and will absorb more water in comparison to freeze-dried pilocarpine HCl tablet. The disintegration medium will penetrate through the pores to initiate shape recovery disintegration mechanisms of the materials (41). The viscosity of the system will increase once the gelatin in contact with the disintegration medium, due to water entrapment within the cross-linking created by hydrogen bonding between the chains of gelatin polymer (42). The entrapment of water means the pores will be filled by bond water and thus limiting the diffusion of gelatin macromolecules and other formulation components into the hydrating medium (42,43). In contrast, the high percentages of highly soluble D-mannitol in the pilocarpine FDT, which were optimised for D-mannitol and gelatin ratio, moderate the viscosity of the system and keep the gelatin chains apart by acting as a bridging molecule and thus enhancing the diffusion of the disintegration medium into the matrix (43,44). Another critical factor to consider is the solubility of the active ingredients in the disintegration medium. The solubility of pilocarpine HCl (the active ingredient of freeze-dried pilocarpine HCl) in water is reported to be 100 mg/mL in comparison to loperamide HCl (the active ingredient of Imodium Instant) which is classified as an active with low solubility profile of 1.02 mg/m (45).

## CONCLUSION

Despite the growing popularity of FDT, there is limited availability of discriminative *in-vitro* disintegration tests currently available. This study aimed to develop a novel and biorelevant disintegration quality control method for FDTs. The system was based on digital imaging technology to provide an opportunity to record precise and frequent disintegration measurements in a bespoke low volume and temperature-controlled vessel to provide an analysis that will discriminate between the disintegration of different types of FDT formulation. The disintegration vessel was 3D-printed, offering flexibility for different pharmaceutical formulations to be tested, facilitating temperature control during the disintegration process (33 and 37°C) and accommodating low volumes of disintegration media (0.05 and 0.7 mL). The 3D-printed disintegration vessel and the disintegration media were loaded into a dual compartment water bath for temperature control. A digital camera attached to a stainless-steel stand, producing a one-unit compact system that was easy to operate. DIDA was sensitive to different tablet disintegration mechanisms poorly observed previously, such as Nurofen Meltlet tablet swelling which can cause a problem for the patient diagnosed with xerostomia in terms of a choking risk. In conclusion, the hypothesis of using digital imaging to evaluate and discriminate between the disintegration of different fast disintegrating tablets was proved using DIDA. The new technique can assess the quality of disintegration; this paper shows that DIDA may differentiate between FDT’s that completely or partially disintegrate, and those which swell rather than disintegrate, in the limited volume of liquid available in the mouth.

## Supplementary Information

ESM 1 (DOCX 457 kb)
