# Supplementary material for: Digital Image Disintegration Analysis: a Novel Quality Control Method for Fast Disintegrating Tablets
Source: AAPS PharmSciTech. 2021 Aug 16;22(7):219. doi: 10.1208/s12249-021-02080-0 (PMC8367883; doi:10.1208/s12249-021-02080-0)
Supplement: Supplementary file 1 — (DOCX 457 kb) [file 12249_2021_2080_MOESM1_ESM.docx]

## Appendix A: Validation of digital camera

Appendix A: Average MGV value of 3D-printed disintegration vessel of freeze-dried pilocarpine HCl tablet and Imodium Instant when it is empty, loaded with 0.7 or 0.05 mL of PBS and with the undissolved tablet. The number of repeats was three for each condition.

## Appendix B: Data Calculation

The MGV gave a measure of the amount of white material in the disintegration vessel. The background MGV, determined of 3D printed disintegration vessel containing only the disintegration medium, was subtracted from all MGVs to eliminate background noise. Calculated MGVs of all images, of a specific disintegration test, were then plotted against time to give the disintegration profile of the test tablet. MGVs were then converted to a percentage of dosage remaining, using the MGV of the full tablet as 100%.


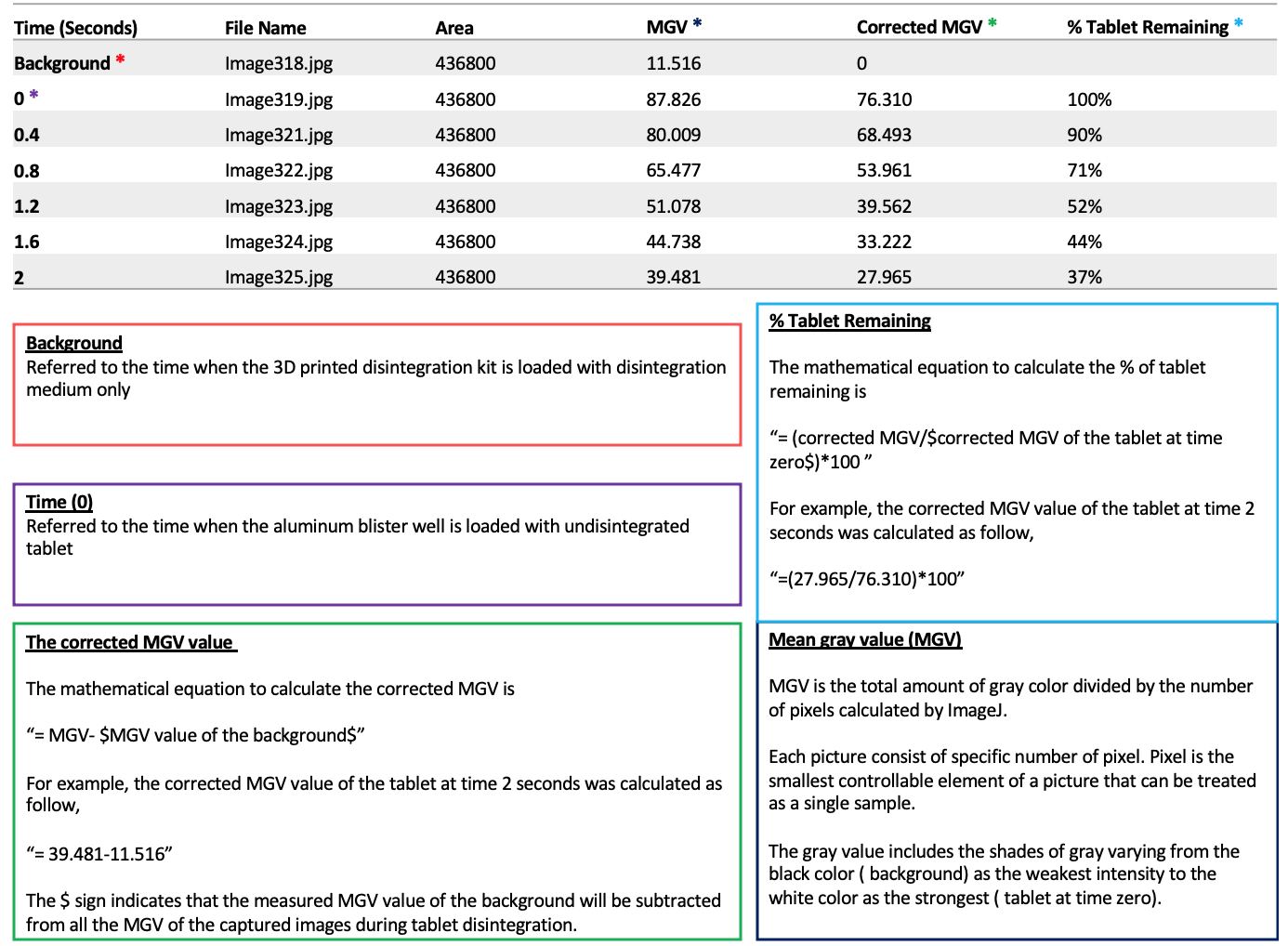


Appendix B: Example of methodology and mathematical equations used to generate disintegration profile.

## Appendix C: Hypromellose tablets

Hypromellose powder (LOT SLBZ2339, Sigma Aldrich), was weighed, 300 mg, and transferred into the tabletting die. The complete set of punches was transferred into the tablet manufacturer, and a force of 5 tonnes was used to prepare the tablet. The handle was unscrewed, and the tablet was removed once the target force had been reached. The manufactured tablet was then placed in a glass vial where 0.7 mL PBS was transferred onto the tablet using a Gilson pipette. The height of the tablet was recorded after 30 minutes of PBS dispensing.


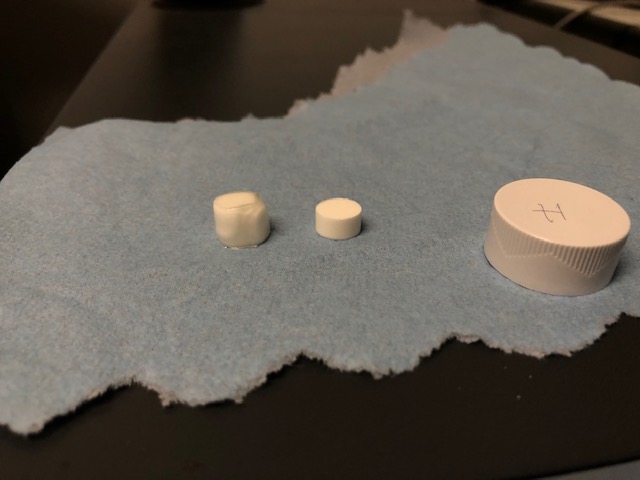

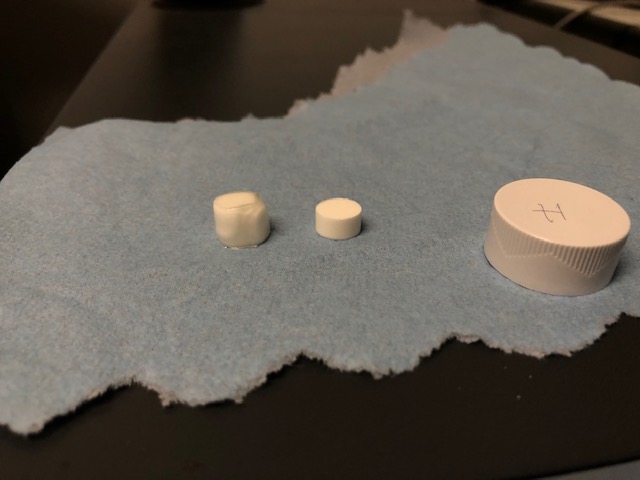


**0 cm**

**0.5 cm**

**0 cm**

**0.7 cm**

Appendix C: Hypromellose tablet, before the addition of PBS (left) and after adding 0.7 mL PBS (right).

Appendix C: The height increases of Hypromellose tablets after the transference of 0.7 mL PBS, n=3.

| Repeats | Height before PBS (cm) | Height after PBS (cm) | Mean Height after PBS (cm) | SD (± cm) | % Height Increase |
| --- | --- | --- | --- | --- | --- |
| 1 | 0.5 | 0.7 | 0.7 | 0.05 | 40 |
| 2 | 0.5 | 0.6 |  |  |  |
| 3 | 0.5 | 0.7 |  |  |  |

## Appendix D: Nurofen Meltlets disintegration time

**The water bath was set at 37 ˚C, using distilled water a beaker was filled to 800 mL. The beaker was inserted into the water bath and allowed to reach to 37 ˚C. The basket-rack assembly was prepared by putting an FDT into each tube and a disc on top of each FDT. The basket was hooked onto the apparatus and the disintegration began. The disintegration was timed for 3 minutes as specified by the British pharmacopoeia (basket apparatus), n=3. Complete disintegration was considered to be completed once all tablet has passed through the mesh.**

| Formulation | Repeats | Observed disintegration time (seconds) | Mean ± SD seconds | % CV |
| --- | --- | --- | --- | --- |
| Nurofen Meltlet | 1 | 20 | 22.3 ± 2.055 | 9 |
|  | 2 | 25 |  |  |
|  | 3 | 22 |  |  |
| Imodium ODT | 1 | 5 | 4.8 ± 0.236 | 5 |
|  | 2 | 4.5 |  |  |
|  | 3 | 5 |  |  |
